# Vitamin E Supplementation Delays Cellular Senescence *In Vitro*

**DOI:** 10.1155/2015/563247

**Published:** 2015-11-03

**Authors:** Giorgio La Fata, Nicole Seifert, Peter Weber, M. Hasan Mohajeri

**Affiliations:** DSM Nutritional Products Ltd., R & D Human Nutrition and Health, P.O. Box 2676, 4002 Basel, Switzerland

## Abstract

Vitamin E is an important antioxidant that protects cells from oxidative stress-induced damage, which is an important contributor to the progression of ageing. Ageing can be studied* in vitro* using primary cells reaching a state of irreversible growth arrest called senescence after a limited number of cellular divisions. Generally, the most utilized biomarker of senescence is represented by the expression of the senescence associated *β*-galactosidase (SA-*β*-gal). We aimed here to study the possible effects of vitamin E supplementation in two different human primary cell types (HUVECs and fibroblasts) during the progression of cellular senescence. Utilizing an unbiased automated system, based on the detection of the SA-*β*-gal, we quantified cellular senescence* in vitro* and showed that vitamin E supplementation reduced the numbers of senescent cells during progression of ageing. Acute vitamin E supplementation did not affect cellular proliferation, whereas it was decreased after chronic treatment. Mechanistically, we show that vitamin E supplementation acts through downregulation of the expression of the cycline dependent kinase inhibitor P21. The data obtained from this study support the antiageing properties of vitamin E and identify possible mechanisms of action that warrant further investigation.

## 1. Introduction

Ageing is a process characterized by gradual functional decline [1, 2] and associated with an increased risk of developing life-threatening diseases [1] such as cancer, diabetes, cardiovascular diseases, and neurodegenerative disorders.

Human aging can be, to some extent, recapitulated* in vitro* using human primary cells which usually undergo a limited number of cellular divisions before reaching a state of irreversible growth arrest termed replicative senescence [3, 4], also known as the “Hayflick Limit” [5, 6]. Moreover, senescent cells become enlarged and lose cell-cell contacts [7, 8].

The changes associated with cellular senescence progression include morphological and biochemical modifications such as increased (and unique) expression of the senescence associated *β*-galactosidase enzyme (SA-*β*-gal) [9] as well as upregulation of the cyclin-dependent kinases (CDKs) inhibitors p16 and p21, which are both negative regulators of cell proliferation [1]. Together, these changes represent important biomarkers used for qualitative and quantitative determination of cellular senescence* in vitro *that is largely accepted to be related to ageing of the organisms* in vivo* [1].

An important contributor to the development of the senescence process is oxidative stress. Oxidative stress usually occurs when the production or the exposure to reactive oxygen species (ROS) overwhelms the antioxidant systems of the cells [10]. ROS are molecules that contain a reactive unpaired electron that can damage biomolecules vital for correct functioning and survival of complex systems [11, 12]. Vitamins E and C are important natural antioxidants capable of neutralizing the deleterious effects of ROS. For this reason and considering their safety, low cost, and the absence of effective alternative treatments, adequate vitamin E and C intake is promoted as a preventive (and potentially curative) treatment for specific pathologies that are typical of old age [12–14].

In this study we monitored the progression of the senescence process in human umbilical vein endothelial cells (HUVECs) and human fibroblasts. We show that the increasing expression of the SA-*β*-gal represents a sensitive and reliable marker to quantify senescent cells in both cellular models. Moreover, we show that addition of vitamin E to the cellular systems is sufficient to reduce the percentage of senescent cells* in vitro*. Lastly, we outline common mechanistic pathways by which vitamin E may exert its antisenescent effect.

## 2. Experimental Section 

### 2.1. Cell Culture

#### 2.1.1. HUVECs

Human umbilical vein endothelial cells (HUVECs) were purchased from Lonza (Basel, Switzerland). Cells were cultured at 37°C, in atmosphere of 5% CO_2_ in Clonetics Endothelial Cell Growth Media (EBM) supplemented with the BulletKit containing bovine brain extract (BBE), epidermal growth factor (hEGF), hydrocortisone, gentamicin, amphotericin B, fetal bovine serum (FBS), and ascorbic acid according to the manufacturer's instructions (Lonza, Basel, Switzerland). Vitamin E (DL-*α*-Tocopherol acetate) (Sigma-Aldrich, Buchs, Switzerland) was added to the growth medium at the final concentration of 25 *μ*M. Cells were passaged before they reached the confluence at a ratio of 1 : 3–1 : 6 according to their proliferating properties over time and at regular intervals. After trypsinization using 0.25% Trypsin/EDTA (Life Technologies Europe, Bleiswijk, Netherlands) cells were seeded at different densities. Cells between passages 3 and 12 were used for the experiments described here.

#### 2.1.2. Fibroblasts

Human dermal fibroblasts were originally isolated from foreskin tissue of a healthy young male subject (DSM Nutritional Products, Kaiseraugst, Switzerland). Fibroblasts were cultured in a humidified incubator at 37°C, 5% CO_2_ in DMEM medium supplemented with 4.5 g/L D-glucose, 4 mM L-glutamine, 100 U/mL penicillin, 100 *μ*g/mL streptomycin (Life Technologies Europe, Bleiswijk, Netherlands), and 10% heat-inactivated FBS (Sigma-Aldrich, Buchs, Switzerland). Subconfluent cells were trypsinized using 0.25% Trypsin/EDTA (Life Technologies Europe, Bleiswijk, Netherlands) and passaged at a ratio of 1 : 3–1 : 5 at regular intervals. Cells between passages 10 and 39 were used for experiments described in this paper.

#### 2.1.3. Vitamin E Supplementation

Vitamin E (DL-*α*-Tocopherol acetate) (Sigma-Aldrich, Buchs, Switzerland) was originally dissolved in 100% EtOH as a stock solution of 50 mM, prediluted 1 : 10 in growth medium, and further diluted 1 : 200 in growth medium to result in a final concentration of 25 *μ*M and a final EtOH concentration of 0.05%. The EtOH concentration was kept constant for all treatment conditions. The vitamin E stock solution in EtOH was stored at −20°C and prepared fresh every week.

### 2.2. Senescence Assessment

#### 2.2.1. SA-*β*-Gal Staining

The senescence state was measured using the SA-*β*-gal kit (Cell Signaling, Beverly, USA) according to the manufacturer's instructions. Briefly, cells were seeded at comparable and nonconfluence density in Costar 48-well plates and cellular senescence was assessed 24 hours (hrs) later. Cells were washed with DPBS and fixed with 2% formaldehyde and 2% glutaraldehyde for 10 min at room temperature (RT). After washing, cells were incubated over night at 37°C in a CO_2_-free incubator with freshly prepared SA-*β*-gal staining solution (1 mg/mL 5-bromo-4-chloro-3-indolyl-*β*D-galactopyranoside in dimethylformamide, 40 mM citric acid/sodium phosphate, 5 mM potassium ferrocyanide, 5 mM potassium ferricyanide, and 150 mM NaCl, 2 mM MgCl_2_) at pH 6.0. Four wells per condition were not stained and used as background control (subtracted from the values of the stained wells). Cellular nuclei were stained with 5 *μ*g/mL Hoechst 33342 solution (Life Technologies Europe, Bleiswijk, Netherlands) for 30 min at RT.

#### 2.2.2. SA-*β*-Gal Automated Analysis

50 adjacent images (fields) of each well were acquired with a 10x objective using an ArrayScan VTI high-content screening system (Thermo Fisher Scientific, Pittsburgh, USA), resulting in a field width of 660 microns. Channel one (Ch1) is the focus channel in which objects (Hoechst-stained nuclei) were identified. SA-*β*-gal staining was detected in Ch2 using the Brightfield module which offers transmitted (white) light illumination for the ArrayScan VTI and subsequently analyzed with the provided Spot Detector BioApplication. Masks were set for measurements within the stained cytoplasmic region by expanding a ring around the nucleus. Dark signal was detected as large spots (cellular debris, vacuoles, or elongated structures associated with refraction of the cell membrane were excluded from the analysis). Reference levels on the “Spot Total Area” feature were used to define a cell as positive (senescent). The percentage of SA-*β*-gal-positive cells was determined as the number of cells developing blue color in their cytoplasm relative to the total number of cells analyzed.

### 2.3. Proliferation

The proliferation rate of the fibroblasts and endothelial cultures was measured using the* in situ* staining for the EdU and Ki67 proliferation markers according to the manufacturer's instructions (see the following). Briefly, cells were seeded at comparable and nonconfluence density in Costar 48-well plates and cellular proliferation was assessed 24 hrs later.

#### 2.3.1. EdU (5-Ethynyl-2′-deoxyuridine) Detection

Cells were incubated with EdU for three hours and fixed in 4% formaldehyde for 15 min at RT. EdU staining was performed according to manufacturer's instructions (Click-iT Plus EdU Alexa Fluor 647 Imaging Kit, Life Technologies Europe, Bleiswijk, Netherlands).

#### 2.3.2. Ki67 Detection

Following EdU staining, also the Ki67 was detected according to manufacturer's instructions (Ki-67 Monoclonal Antibody, Mouse (7B11), FITC Conjugate, Life Technologies Europe, Bleiswijk, Netherlands). Incubation with Ki-67 antibody was performed for 4 hrs at RT.

#### 2.3.3. Proliferation Analysis

Images of three separate fluorescent channels (Hoechst, FITC, and Deep Red) were acquired on the ArrayScan VTI high-content screening system (Thermo Fisher Scientific, Pittsburgh, USA) using a 10x objective with suitable filter sets. Hoechst 33342 was used to stain and identify the nuclei. 50 fields per well were counted using object selection parameters and a fluorescence intensity threshold to select only Ki67 and EdU positive cells. Image analysis was performed using the provided Cell Health Profiling BioApplication; the critical well-level output parameters reported the percentage of selected cells for each channel.

### 2.4. Real Time Polymerase Chain Reaction (PCR)

#### 2.4.1. RNA Isolation

Cells were seeded in a 6-well plate and cultured as already described. Three days after seeding, cells were washed in DPBS and RNA was extracted using the RNeasy Mini Kit (Qiagen, Hombrechtikon, Switzerland) according to manufacturer's instructions. Quantity and quality of the RNA were assessed using the Nanodrop ND-1000 Spectrophotometer (Thermo Scientific, USA) and the Agilent 2100 Bioanalyzer (Agilent Technologies, Basel, Switzerland), respectively, according to manufacturer's instructions.

#### 2.4.2. RT-PCR

Reverse transcription was performed using the Omniscript RT Kit with 2 *μ*g of RNA according to the manufacturer's instructions (Qiagen, Hombrechtikon, Switzerland). Each PCR was performed in triplicate using 40 ng of cDNA and the 7900 HT real time PCR system (Applied Biosystems, Foster City, California, USA). All values were normalized to the expression of the 18S ribosomal RNA gene (internal standard). The sequences of the primers and probes used are the following:

 
*Human P21* (NM_000389.4): Forward 5′-TGGAGACTCTCAGGGTCGAAA-3′; Reverse 5′-GGCGTTTGGAGTGGTAGAAATC-3′; Probe (FAM conjugated): 5′-CGGCGGCAGACCAGCATGAC-3′.
 
*Human P16* (NM_00077.4): Forward 5′-CATAGATGCCGCGGAAGGT-3′; Reverse 5′-AAGTTTCCCGAGGTTTCTCAGA-3′; Probe (FAM conjugated): 5′-CCTCAGACATCCCCGATTGAAAGAACC-3′.
 
*Human 18S* (NR_003286.2): Forward 5′-CGGCTACCACATCCAAGG-3′; Reverse 5′-CGGGTCGGGAGTGGGT-3′; Probe (VIC conjugated): 5′-TTGCGCGCCTGCTGCCT-3′.

Fold changes were measured as follows: 2^−ddCt^ (ddCt = dCt each value – dCt baseline (younger stage CTR); dCt = Ct value target gene – Ct value internal standard (18S). All fold change values were shown as relative to the younger stage and control (CTR) condition that was indicated as 100% of gene expression.

## 3. Results

### 3.1. SA-*β*-Gal: Marker of Cellular Senescence

**Figure 1:**
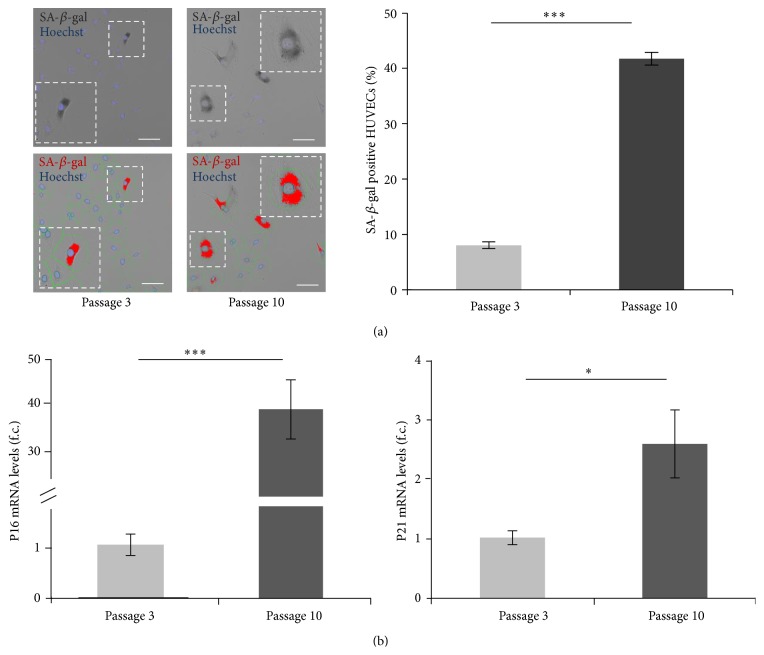
Comparison of HUVEC cultures at early and late passage. (a) Representative images of HUVEC cultures stained for the SA-*β*-gal (upper panels, dark grey cells) at passage 3 and passage 10. Lower panels show the digital images used to quantify the % SA-*β*-gal positive cells (red color; see materials and methods for details). Nuclei are stained with Hoechst (blue). Scale bars = 100 *μ*m. Graph: percentage of SA-*β*-gal positive cells. *N* = 8 for both analyses. *t* test, *p* < 0.001  (^*∗∗∗*^). Error bars indicate the standard error mean (s.e.m.). (b) Graphs show the expression levels of the P16 (left) and P21 (right) genes measured in HUVEC cultures. Values indicate the fold change (f.c.). All values were normalized to the expression levels of the housekeeping gene 18S. Error bars represent the s.e.m. For both analyses *n* = 4. P16 passage 3 = 1.1, passage 10 = 38.6. *t* test: *p* < 0.001  (^*∗∗∗*^); P21 passage 3 = 1.0, passage 10 = 2.6. *t* test: *p* = 0.035  (^*∗*^).

To verify that the SA-*β*-gal quantification could be used as a reliable marker of cellular senescence, we tested our two* in vitro *models at different passages. Using an automated analysis system, we detected a significantly (*p* < 0.001) increased percentage (%) of SA-*β*-gal positive cells in HUVEC cultures at passage 10 (42%) when compared with younger ones at passage 3 (8%) (Figure 1(a)). Passage 10 HUVECs also showed larger nuclei (299 *μ*m^2^ versus 277 *μ*m^2^ for cultures at passage 3) (*p* = 0.007), abnormal morphology (bigger cell surface, flatter cells, and less cell-cell contacts) (Supplementary Figure 1(a)) (see Supplementary Material available online at http://dx.doi.org/10.1155/2015/563247), and consistently longer doubling time (cultures at early passages = 1.8 to 2.1 days versus cultures at late passages = 2.7 to 3.9 days) as well as significant reduced percentage of EdU positive cells (passage 10 cultures: 15% versus passage 3 cultures: 31%) (*p* < 0.001) (Supplementary Figure 1(b)) and Ki67 positive cells (passage 10 cultures: 23% versus passage 3 cultures: 57%) both used as markers of cellular proliferation. EdU (5-ethynyl-2′-deoxyuridine) is a nucleoside analog of thymidine incorporated into DNA during active DNA synthesis, while Ki67 is a protein that is expressed in the nucleus during interphase and therefore strictly associated with cell proliferation [15, 16]. Similar results were also obtained in human fibroblasts where the significant higher percentage of SA-*β*-gal positive cells (*p* < 0.001) was detected in cultures at passage 31 (31%) when compared to younger ones at passage 15 (10%) (Supplementary Figure 2(a)). Moreover, in this cellular model, morphological (Supplementary Figure 2(a)) and proliferation differences were detected during the progression of senescence (passage 15 cultures: 57% and 49% Ki67 and EdU positive cells, resp.; passage 31 cultures: 26% and 21% Ki67 and EdU positive cells, resp.) (Supplementary Figure 2(c)).

### 3.2. Expression of the P21 and P16 Genes in HUVEC and Human Fibroblast Cultures at Early and Late Passage

The morphological and behavioral changes occurring during the progression of cellular senescence are inevitably associated with changes in the expression of specific genes. Several studies have shown an increased protein expression of the two CDKs inhibitors, p21 and p16, in senescent human fibroblasts [17–19] as well as in senescent HUVECs [4]. Therefore, changes in the expression of both of these proteins are considered markers of senescence.

To verify that these genes were also differently expressed in our cellular models, we measured their expression levels using real time polymerase chain reaction (RT-PCR). HUVEC cultures with a large number of senescent cells (refer to Figure 1) showed an increased expression of both the P16 and P21 genes when compared with cultures having lower percentage of SA-*β*-gal positive cells (*p* < 0.001 and *p* = 0.035, resp.) (Figure 1(b)). Similar results were also observed when comparing the P21 expression in fibroblasts, although the P16 expression was not increased (Supplementary Figure 3). Of note in HUVECs, the induction of P16 expression was much higher than P21 induction (Figure 1(b): comparing fold changes).

Therefore these experiments established that the measurement of SA-*β*-gal positive cells represents a reliable marker to quantify senescence in HUVECs and human fibroblasts.

### 3.3. Effect of Vitamin E Acute Supplementation on Senescence and Proliferation

We tested whether supplementation with the antioxidant compound vitamin E could reduce the number of senescent cells that appeared* in vitro* over time (refer to Figure 1).

**Figure 2:**
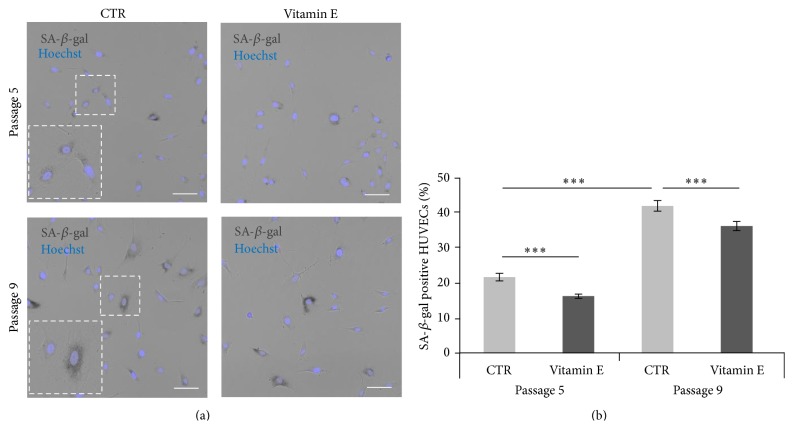
Acute vitamin E supplementation reduces the percentage of SA-*β*-gal positive HUVECs. (a) Representative images of the graph shown (b) comparing HUVEC cultures at passages 5 and 9 stained for the SA-*β*-gal (dark grey cells). Enlargements highlight the morphological differences observed comparing early and late passages. Nuclei are visualized in blue. Scale bars = 100 *μ*m. (b) Graph showing the percentage of SA-*β*-gal positive cells measured in passage 5 and passage 9 HUVEC cultures. For all analyses *n* = 8. Passage 5: CTR = 22%, vitamin E = 16%. *t* test *p* = 0.001  (^*∗∗∗*^); passage 9: CTR = 42%, vitamin E = 36%. *t* test, *p* = 0.001  (^*∗∗∗*^); CTR (passage 5) versus CTR (passage 9) cultures: *t* test, *p* < 0.001  (^*∗∗∗*^). Error bars represent the s.e.m.

HUVEC cultures at passages 5 and 9 grown in optimal conditions (see materials and methods) were supplemented with vitamin E (25 *μ*M) for 24 hrs and the percentage of SA-*β*-gal positive cells was measured (Figure 2). As expected, the number of SA-*β*-gal positive cells significantly (*p* < 0.001) increased at later passages (Figure 2(b): comparing percentage of SA-*β*-gal positive cells in control (CTR) of passage 5 cultures = 22% with CTR of passage 9 cultures = 42%). Importantly, 24 hrs vitamin E treatment was sufficient to significantly reduce (*p* < 0.001) the number of SA-*β*-gal positive cells at both stages (CTR = 22% versus vitamin E = 16% and CTR = 42% versus vitamin E = 36%, resp.) (Figure 2(b); see the caption of Figure 2 for details).

**Figure 3:**
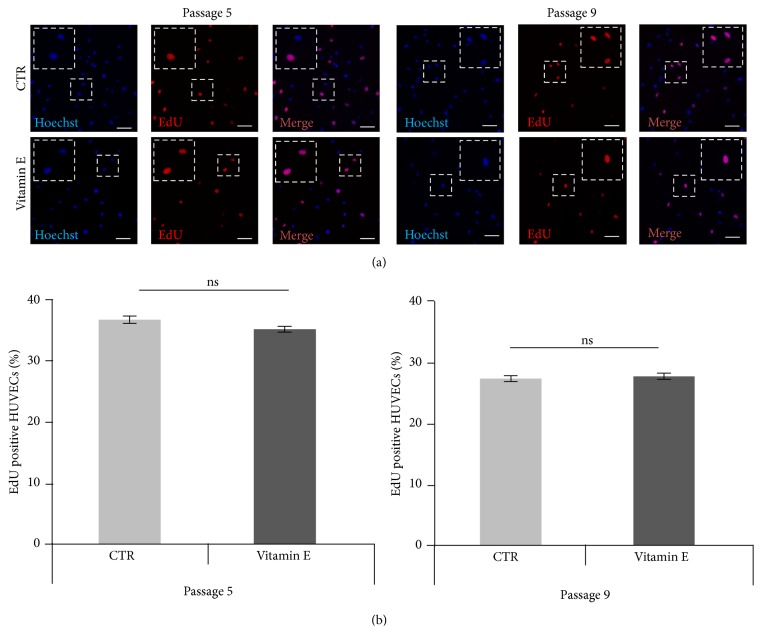
Vitamin E treatment does not alter the percentage of proliferating HUVECs. (a) Representative images showing passage 5 and passage 9 (CTR and vitamin E treatment) HUVEC cultures stained for the proliferation marker EdU (red). Nuclei are visualized by Hoechst staining (blue). Scale bars = 100 *μ*m. (b) Graphs represent the percentage of EdU positive HUVECs. Error bars represent s.e.m. For all analyses *n* = 8. Passage 5: CTR = 37%, vitamin E = 35%. *t* test: *p* > 0.05 (ns). Passage 9: Ctrl = 27%, vitamin E = 27%. *t* test: *p* > 0.05 (ns).

Senescent cells are characterized by irreversible proliferation arrest [1, 5, 20]; therefore we verified whether the reduced number of SA-*β*-gal positive cells following vitamin E treatment was also associated with an increased proliferating rate of the HUVECs cultures. 24 hrs after vitamin E treatment no difference, in the number of dividing cells (EdU positive cells), was observed neither in the early passages nor in the late passages (Figure 3). Similar results were observed when the percentage of Ki67 positive cells was analyzed (Supplementary Figure 4). As expected, a reduced number of cells positive for both of these proliferation markers were measured at later passages (Figure 3 and Supplementary Figure 4).

**Figure 4:**
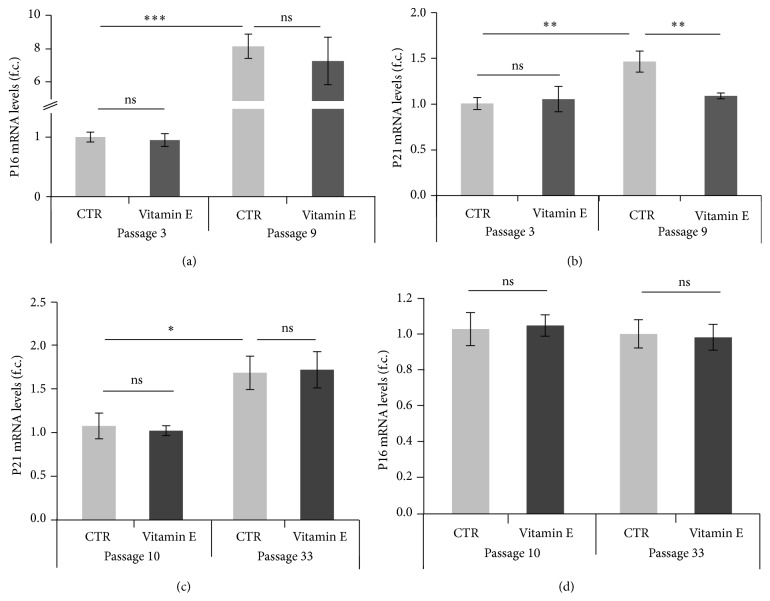
Acute vitamin E treatment downregulates the expression of the P21 gene in HUVEC cultures at passage 9. Gene expression analysis of HUVEC and human fibroblasts cultures at different passages and following vitamin E treatment. Error bars represent s.e.m. All values were normalized to the expression of the housekeeping gene 18S. Gene expression is indicated as fold change (f.c.). (a) P16 gene expression analysis in HUVECs. Passage 3: CTR = 1.0 *n* = 3, vitamin E = 1.0 *n* = 4; *t* test: *p* > 0.05 (ns). Passage 9: CTR = 8.5, *n* = 4, vitamin E = 7.7, *n* = 4; *t* test, *p* > 0.05 (ns). Passage 3 CTR versus passage 9 CTR: *t* test, *p* < 0.001  (^*∗∗∗*^). (b) P21 gene expression in HUVECs. Passage 3: CTR = 1.0 (*n* = 4), vitamin E = 1.1 (*n* = 4); *t* test: *p* > 0.05 (ns). Passage 9: CTR = 1.5 (*n* = 3), vitamin E = 1.1 (*n* = 4); *t* test, *p* = 0.015  (^*∗∗*^). Passage 3 CTR versus passage 9 CTR: *t* test, *p* = 0.014  (^*∗∗*^). (c) P21 gene expression in human fibroblasts. For all analyses *n* = 8. CTRs were already reported and described in Figure 2. Passage 10: CTR = 1.1, vitamin E = 1.0; *t* test: *p* > 0.05 (ns). Passage 33: CTR = 1.7, vitamin E = 1.7; *t* test, *p* > 0.05 (ns). (d) P16 gene expression in human fibroblasts. For all analyses *n* = 8. CTRs were already reported and described in Figure 2. Passage 10: CTR = 1.0, vitamin E = 1.0; *t* test: *p* > 0.05 (ns). Passage 33: CTR = 1.0, vitamin E = 1.0; *t* test, *p* > 0.05 (ns).

Given the reduction in SA-*β*-gal positive cells following vitamin E treatment, we next examined whether the expression levels of genetic markers of senescence were also affected. Using RT-PCR we measured the expression levels of the P16 and P21 genes in HUVEC cultures (at passages 3 and 9) grown in optimal conditions and supplemented with 25 *μ*M of vitamin E. In agreement with previous results (Figure 1), we measured a significant overexpression of the P16 (*p* < 0.001) and P21 (*p* = 0.01) genes when passage 9 cultures were compared to passage 3 cultures (Figures 4(a) and 4(b)). When the vitamin E effect was analyzed, we detected downregulation of P21 gene expression (*p* = 0.015) in passage 9 HUVEC cultures but not in early passage cultures (Figure 4(b)) but detected no effect regarding the P16 expression (Figure 4(a)). Similar results were also obtained in human fibroblasts where, surprisingly, an increased expression of the P21 gene (CTR passage 10 versus CTR passage 33) was observed over time (Figure 4(c)) but not overexpression of the P16 gene (Figure 4(d)). In this case instead, acute vitamin E treatment did not affect the expression of the analyzed genes.

Therefore, acute vitamin E supplementation reduces the number of SA-*β*-gal positive HUVECs without affecting the proliferation rate and furthermore vitamin E supplementation downregulates the overexpression of the P21 gene observed in older HUVEC cultures.

### 3.4. Effect of Chronic Vitamin E Supplementation in HUVECs

As acute vitamin E treatment reduces the percentage of SA-*β*-gal positive cells in both passage 5 and 9 HUVEC cultures (Figure 2) and P21 expression is downregulated following acute vitamin E treatment (Figure 4(b)), we decided to test if a prolonged (chronic) vitamin E supplementation was more effective in reducing the senescence parameters associated with ageing* in vitro*.

**Figure 5:**
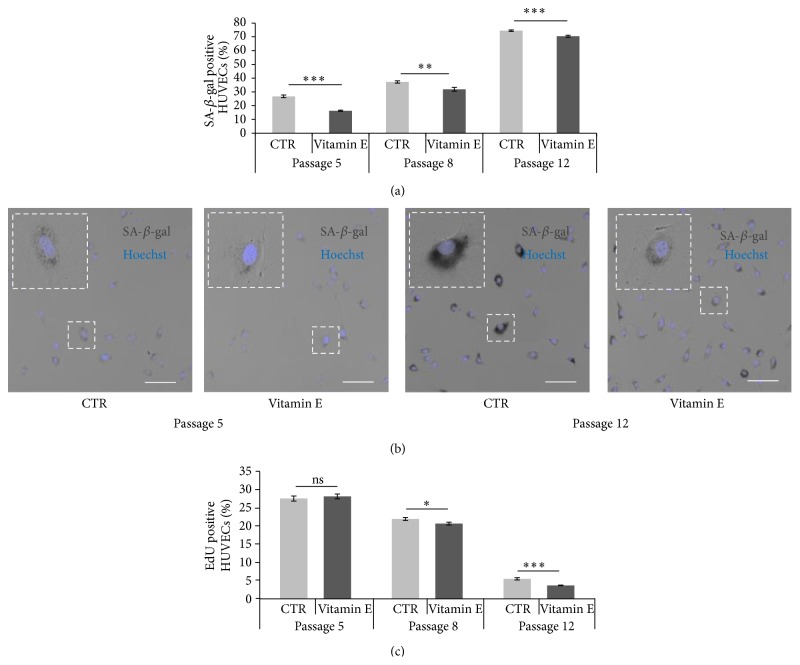
Chronic vitamin E supplementation reduces the percentage of SA-*β*-gal positive and proliferating HUVECs. (a) Graph showing the percentage of SA-*β*-gal positive cells measured in HUVEC cultures at passages 5, 8, and 12. For all conditions *n* = 8. Error bars represent the s.e.m. Passage 5: CTR = 27%, vitamin E = 16%; *t* test, *p* < 0.001  (^*∗∗∗*^); passage 8: CTR = 37%, vitamin E = 32%; *t* test, *p* = 0.003  (^*∗∗*^); passage 12: CTR = 74%, vitamin E = 70%; *t* test, *p* < 0.001  (^*∗∗∗*^). (b) Representative images of the graph shown in (a) comparing passage 5 and passage 12 HUVEC cultures stained for the SA-*β*-gal (dark grey cells). Enlargements highlight the morphological differences. Nuclei are visualized in blue (Hoechst staining). Scale bars = 100 *μ*m. (c) Graph showing the percentage of EdU positive cells measured in HUVEC cultures at passages 5, 8, and 12. Error bars represent the s.e.m. For all analyses *n* = 10. Passage 5: CTR = 27.6%, vitamin E = 28.2%; *t* test, *p* > 0.05 (ns); passage 8: CTR = 22%, vitamin E = 20.7%; *t* test, *p* = 0.038  (^*∗*^); passage 12: CTR = 5.5%, vitamin E = 3.7%; *t* test, *p* < 0.001  (^*∗∗∗*^).

HUVECs were cultured in optimal conditions and supplemented with vitamin E starting from an early passage. Cultures were maintained* in vitro* until most of the cells were considered senescent due to the following criteria: decreased cell division rate and adaptation of an abnormal morphology typical of senescent cells (refer to Figure 1 and Supplementary Figure 1). As expected, we measured an increase in the number of SA-*β*-gal positive cells in the cultures over time (Figures 5(a) and 5(b) compare CTRs at different passages). In agreement with the acute treatment data, the long-term vitamin E supplementation showed a constant and significant reduction of the percentage of SA-*β*-gal positive cells over time (Figures 5(a) and 5(b)). Interestingly, age-dependent increase of SA-*β*-gal positive cells and a similar vitamin E effect were also measured in human fibroblasts (Supplementary Figure 5).

The proliferation rate (percentage of EdU positive cells) of these cultures exhibited no differences at early stages but significantly differed when the cultures were older (Figure 5(c)) suggesting that vitamin E chronic treatment reduced the number of SA-*β*-gal HUVECs by reducing their proliferative capacity and therefore delaying the senescence onset.

**Figure 6:**
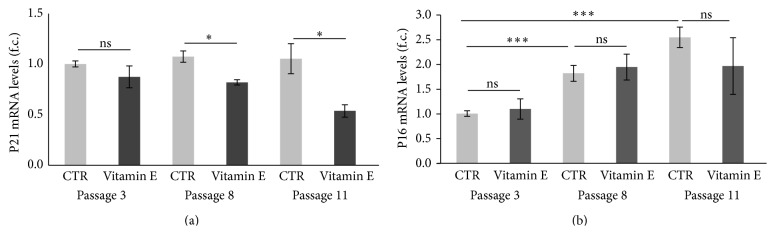
Chronic vitamin E supplementation reduces the expression levels of the P21 gene. P21 and P16 gene expression analysis in passage 5, 8, and 11 HUVEC cultures chronically treated with vitamin E. Error bars represent s.e.m. All values were normalized to the expression of the housekeeping gene 18S. Gene expression is indicated as f.c. Unless differently indicated, *n* = 5. (a) P21 gene expression. Passage 5: CTR = 1.0, vitamin E = 0.9; *t* test: *p* > 0.05 (ns). Passage 8: CTR = 1.1, vitamin E = 0.8; *t* test, *p* = 0.03  (^*∗*^). Passage 11: CTR = 1.0; vitamin E = 0.5, *n* = 3; *t* test: *p* = 0.04  (^*∗*^). (b) P16 gene expression. Passage 5: CTR = 1.0, vitamin E = 1.1; *t* test: *p* > 0.05 (ns). Passage 8: CTR = 1.8, vitamin E = 1.9; *t* test: *p* > 0.05 (ns). Passage 11: CTR = 2.5; vitamin E = 2.0, *n* = 3; *t* test: *p* > 0.05 (ns). CTR passage 5 versus passage 8 and passage 11: *t* test, *p* < 0.001  (^*∗∗∗*^).

Finally we also measured the expression levels of the P21 and P16 genes and found that in agreement with our previous data (refer to Figure 4) chronic vitamin E treatment decreased the expression levels of the P21 gene but not the expression of P16 (Figure 6). Of note, during this long-term treatment a less pronounced overexpression of the P16 gene was detected in later passages (Figure 6(b)) when compared to earlier ones (2.5-fold change versus 8-fold change observed previously: refer to Figure 4). Moreover, no age effect was in this case observed when the P21 gene was analyzed (Figure 6(a) compares CTRs at different passages). Such apparent inconsistencies may be justified by the experimental conditions inherent in chronic versus acute treatment regimes.

## 4. Discussion 

The data presented here provide evidence for the antisenescence activity of vitamin E* in vitro*. Antioxidant vitamins are known to protect cells from oxidative stress, a major contributor to ageing [12, 21–23]. Here we modeled human ageing* in vitro* by continuously culturing primary cells until they reached a state of irreversible growth arrest, also known as cellular senescence.

The quantification of cellular senescence adopted in this study is based on the automatic detection of the SA-*β*-gal positive cells present in a culture at a given time. Despite the extensive utilization of the SA-*β*-gal staining to determine the senescent status of cells* in vitro *[9], limited studies report that this assay is not robust and reproducible enough for a quantitative analysis [24–26]. Therefore, we first tested whether the SA-*β*-gal quantification could be used as a reliable marker of cellular senescence in our two* in vitro *models at different passages. Human endothelial cells (HUVECs) were utilized in this report and compared with human fibroblasts, the most common cellular model to study senescence. In agreement with previous reports [4, 17, 19, 27, 28] we observe a significant increase of SA-*β*-gal positive cells, including altered cell morphology, reduced proliferation rate, and overexpression of the P16 and P21 genes in HUVEC cultures at late passages (Figure 1 and Supplementary Figure 1). Although a combination of many biomarkers for senescence is preferred [6], our experimental method is quantitative and reliable enough to measure the senescence state* in vitro*. This conclusion is also supported by our data obtained in other human primary cells (human fibroblasts) where an increased number of SA-*β*-gal positive cells are measured in association with altered morphology and reduced proliferation rate (Supplementary Figure 2). Of note, in agreement with [4] and differently from what described in HUVECs [29, 30], human fibroblasts (analyzed at late passages) do not show an increased size of the nuclei (Supplementary Figure 2).

Considering that adequate vitamin E intake was previously associated with a reduction in cognitive decline over time [12, 14, 31, 32] as well as improvement of specific symptoms in individuals affected by Alzheimer's disease (AD) [12, 33, 34], we tested vitamin E effect on ageing* in vitro*. Using the quantification of SA-*β*-gal positive cells, we show here that acute vitamin E supplementation is sufficient to reduce the number of senescent HUVECs at early and late passages (Figure 2). These results are in agreement with Makpol et al. who reverted the senescence phenotype of human fibroblasts using specific vitamin E isomers (tocotrienols) [35]. To further validate this vitamin E effect, we supplemented HUVEC cultures with vitamin E starting from early passages and for the entire culturing time (chronic treatment). The experiment was concluded only when the cultures where dividing very slowly and showed a number of SA-*β*-gal positive cells over 60%. In this case vitamin E treatment resulted in a significant and consistent reduction of the number of SA-*β*-gal positive HUVECs (Figure 5). A similar phenotype was also described for human fibroblasts (Supplementary Figure 5). In both of our experimental systems, the progression of senescence was reduced but not arrested during “ageing” suggesting that vitamin E delays the onset of senescence.

Our data that vitamin E reduces senescence in aged cultures is in agreement with epidemiological human data. Nutrient intakes were assessed with a 146-item food-frequency questionnaire in a cross-sectional study (586 participants, aged 35–74 y), and relative telomere length of leukocyte DNA was measured. After adjustment for age and other potential confounders, multivitamin use was associated with longer telomere length. In the analysis of micronutrients, higher intakes of vitamins C and E from foods were each associated with longer telomeres, even after adjustment for multivitamin use [36]. Telomere length has been proposed as a marker of biological ageing because telomeres typically shorten by a few dozen to a couple hundred base pairs (bps) per cell division eventually leading to chromosomal instability, senescence, and cell death [37]. Therefore, these human data strongly suggest an antiageing effect of vitamins C and E in humans. Considering the existing inverse correlation between senescence and proliferation, we then verified whether vitamin E treatment altered the proliferation rate of cultures during senescence. We measured the number of EdU and Ki67 positive cells as markers of proliferation [15, 16] and found that vitamin E does not influence this process in early or late passage cells after acute treatment (Figure 3 and Supplementary Figure 4). Conversely, chronic supplementation of HUVECs cultures showed differences in the proliferation rate following vitamin E treatment at later passages (Figure 5(c)). These data suggest that, under optimal conditions, prolonged vitamin E supplementation reduces senescence progression through mechanisms that alter cellular proliferation.

When the possible effects of vitamin E supplementation were monitored at molecular level, we found a vitamin E-dependent reduction of the elevated expression of the P21 gene in HUVEC cultures at late passages (Figure 4) (acute treatment). This effect was also conserved under chronic treatment conditions (Figure 6). A similar effect was not observed for P16 gene expression in both HUVECs and human fibroblasts. It is possible that the fibroblast cultures contained too few senescent cells to detect such an effect associated with vitamin E. Indeed, although overexpression of the P21 gene was measured in fibroblast at passage 33, similar overexpression was not detected for the P16 gene (Figures 4(c) and 4(d)); perhaps due to different kinetic these genes are subjected to during senescence progression [1]. Another possible reason may be associated with a difference in the senescence program that HUVECs and fibroblasts follow [4] and/or differences in intrinsic factors provided in the culturing media.

The P21-dependent effect of vitamin E observed in our study is in line with mechanistic data* in vivo* showing that long-term dietary vitamin E (alpha-tocopherol) supplementation increases the lifespan of wild-type mice [38]. The authors argue that the increase in lifespan, referred to as an antiageing effect, may reflect an anticancer process that occurs via the induction of the P21 signaling pathway [38].

In conclusion, our data demonstrate that through specific micronutrient supplementation it is possible to delay the onset of cellular senescence in two* in vitro* models: human endothelial cells and fibroblasts. Moreover, our data suggest that these compounds may act through the P21 pathway. Additional research is needed to identify the mechanisms that drive and regulate cellular senescence and to identify specific pathways that can be regulated by supplementation with particular micronutrients.

## 5. Conclusions 

In this study we show that vitamin E supplementation reduces the number of SA-*β*-gal positive cells* in vitro*. Our data are in favor of reduced cellular senescence associated with the activity of this compound that is already used as preventive and curative measures for specific pathologies typical of old age. More research is needed to study the mechanisms by which vitamin E intake can ameliorate ageing and extend longevity.

## Supplementary Material

Supplementary Figure 1 compares the nuclei size and proliferation rate of HUVEC cultures at passage 3 and 10. Supplementary Figure 2 compares the phenotype and the behavior of human fibroblasts at early and late passages. Supplementary Figure 3 describes the gene expression analysis (for the P16 and P21 mRNAs) performed in human fibroblasts at early and late passages. Supplementary Figure 4 shows the proliferation analysis using Ki67 as a marker for proliferation. Supplementary Figure 5 shows the effect of a chronic vitamin E supplementation in human fibroblasts during the progression of cellular senescence.
