# Supplementary material for: Vitamin E Supplementation Delays Cellular Senescence In Vitro
Source: Biomed Res Int. 2015 Nov 3;2015:563247. doi: 10.1155/2015/563247 (PMC4647025; doi:10.1155/2015/563247)

Supplementary Materials

Supplementary Figure 1: Comparison of HUVEC cultures at early and late passage.
a) Representative images showing the abnormal cell morphology and increased nuclei size of cells at late passage. Scale bars=100µm.
Graph: average nuclei size (µm^2). Ttest p=0.007 (**).
b) Representative images showing cells stained for the proliferation marker EdU (red) and Hoechst (nuclei, blue).
Graph: % of EdU positive cells. N=8 for both analysis. Ttest P<0.001 (***).

Supplementary Figure 2: Comparison of human fibroblasts at different passages.
a) Left graph representing the % of SA-β-gal positive fibroblasts in cultures at passage 15 and 31. Ttest p<0.001 (***), n=8, error bars represent s.e.m. Right graph represents the nuclei size: Ttest p>0.05 (ns), n=12, error bars represent s.e.m.
b) Representative images of human fibroblasts stained for the SA-β-gal (green/blue color). Morphological details are enlarged. Scale bars=200µm.
c) Proliferation rate of fibroblasts at passage 15 and 31, measured using the Ki67 and EdU proliferation markers. Ttest p<0.001 (***), n=8, error bars represent s.e.m.

Supplementary Figure 3: P16 and P21 mRNA expression levels in human fibroblast cultures.
Graphs show the expression levels for the P16 (left) and P21 (right) genes measured in human fibroblast cultures at passage 10 and 33. Values indicate the fold change (f.c.). All values were normalized to the expression levels of the housekeeping gene 18S. Error bars represent the s.e.m. For both analysis n=4.
P16 passage 10=1.0, passage 33=1.0. Ttest: p>0.05 (not statistically significative: ns); P21 passage 10=1.1, passage 33=1.7. Ttest: p=0.02 (*).

Supplementary Figure 4: Vitamin E treatment does not alter the % of proliferating HUVECs.
a) Representative images showing passage 5 and passage 9 (CTR and vitamin E treatment) HUVEC cultures stained for the proliferation marker Ki67 (green). Nuclei are visualized by Hoechst staining (blue). Scale bars=100µm.
b) Graphs represent the % of Ki67 positive HUVECs. For all conditions n=8, Ttest: p>0.05 (ns). Error bars represent s.e.m. Passage 5: CTR=55%, vitamin E=52%. Passage 9: CTR=34%, vitamin E=33%.

Supplementary Figure 5: Chronic vitamin E supplementation reduces the % of SA-β-gal positive fibroblasts.

Graph showing the % of SA-β-gal positive cells measured in fibroblasts cultures at passages 20, 34, 36, 39. For all conditions n=8. Error bars represent the s.e.m.

Passage 20: CTR=5.2%, vitamin E=5.9%, Ttest $p>0.05$ (ns);

Passage 34: CTR=13.1%, vitamin E=8.1%, Ttest $p<0.001$ (***);

Passage 36: CTR=31.5%, vitamin E=9.2%, Ttest $p<0.001$ (***);

Passage 39: CTR=61.7%, vitamin E=54.9%, Ttest $p<0.001$ (***).

Supplementary Figure 1: Comparison of HUVEC cultures at early and late passage.

a)

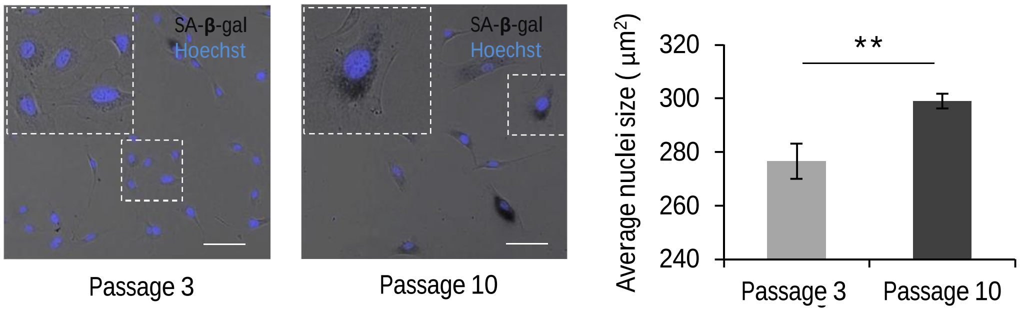

b)

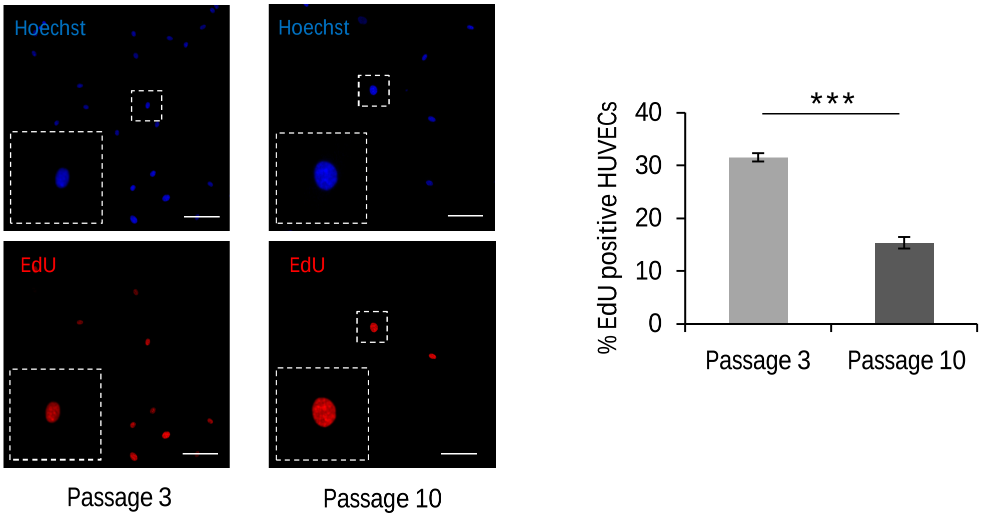

Supplementary Figure 2: Comparison of human fibroblasts at early and late passage.

a)

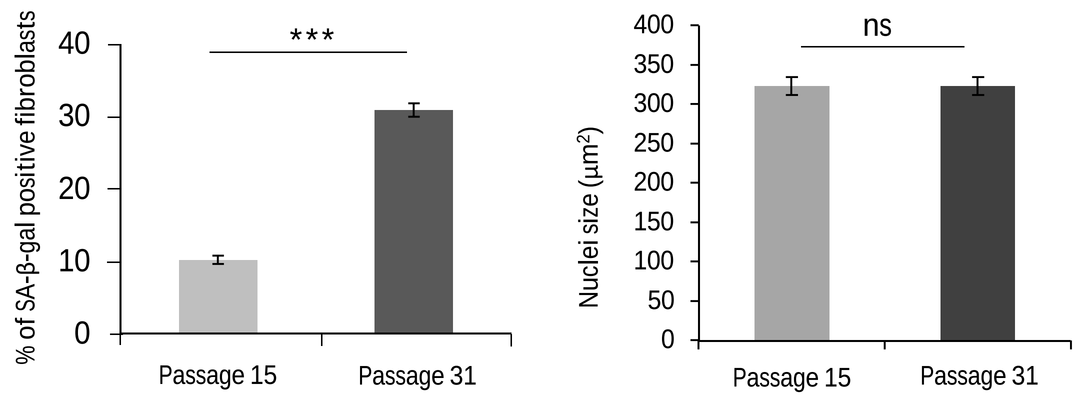

b)

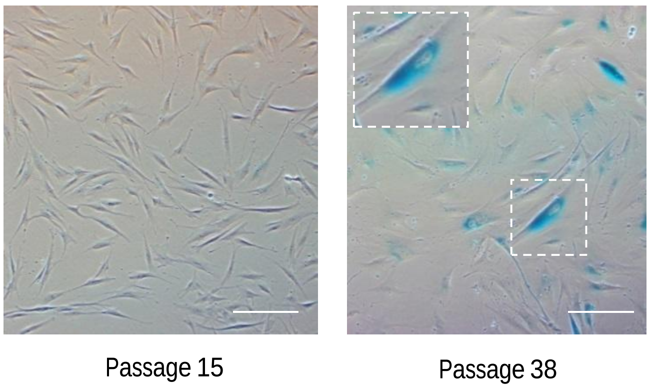

c)

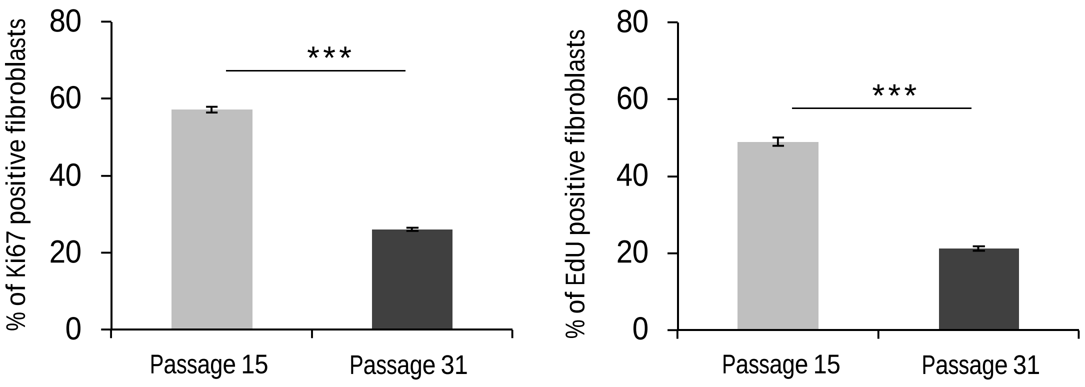

Supplementary Figure 3: P16 and P21 mRNA expression levels in human fibroblast cultures.

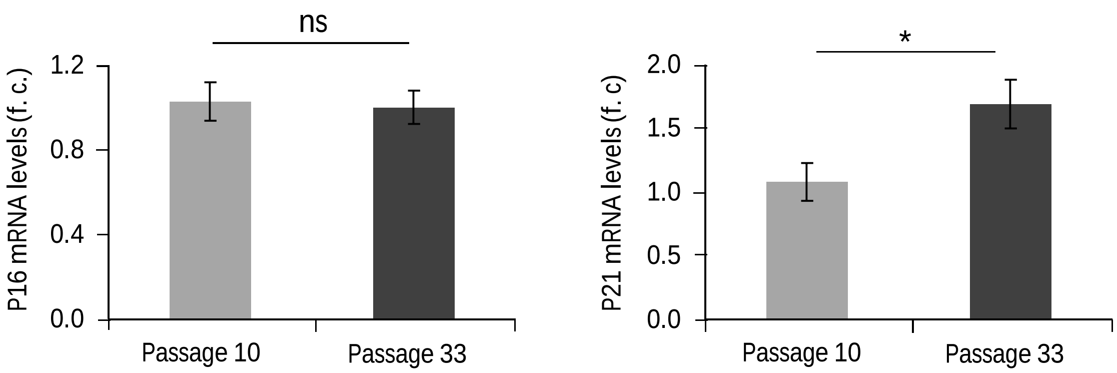

Supplementary Figure 4: Vitamin E treatment does not alter the % of proliferating HUVECs.

a)

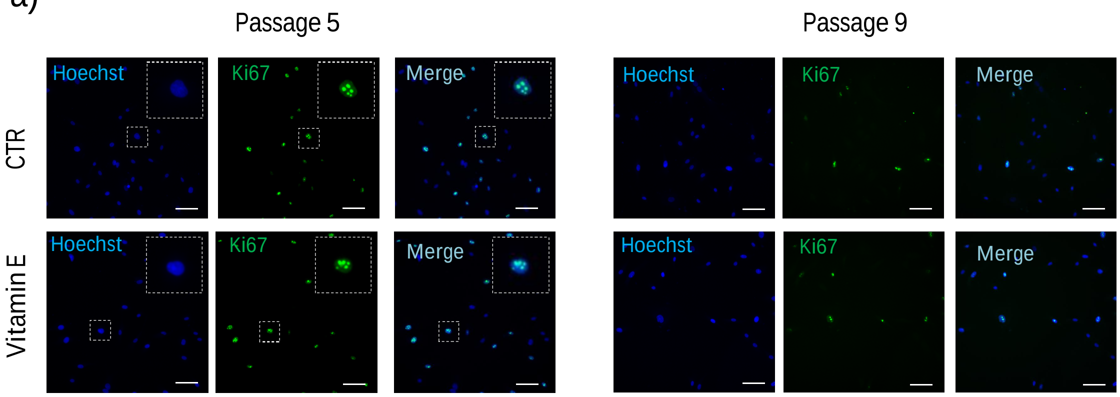

b)

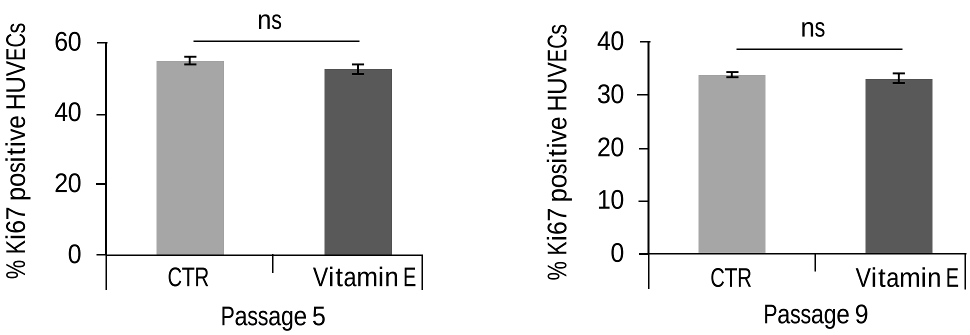

Supplementary Figure 5: Chronic vitamin E supplementation reduces the % of SA-**β**-gal positive fibroblasts.

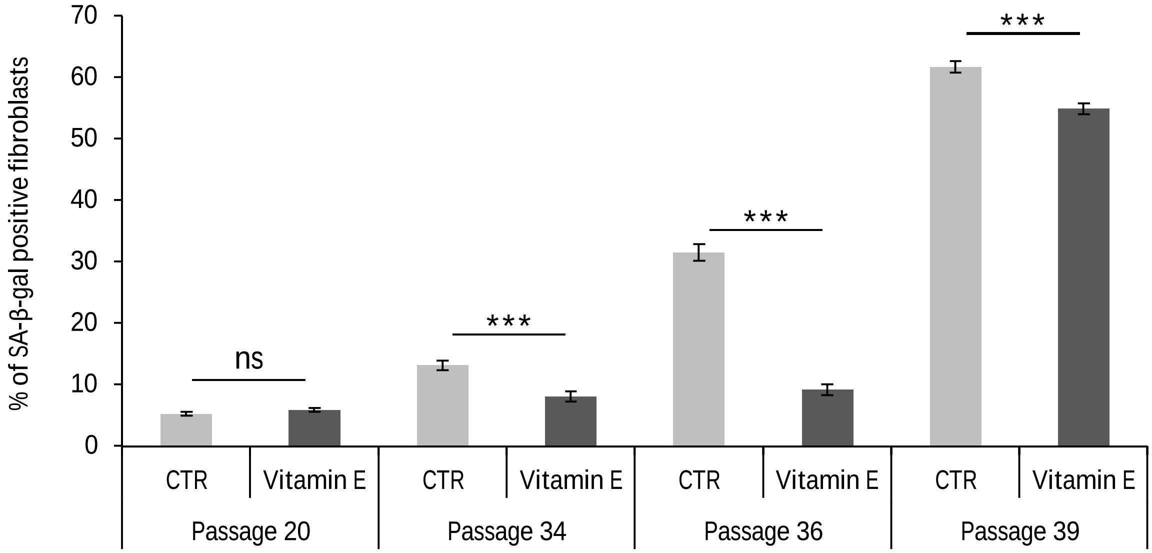

Supplement: Supplementary file 1 — Supplementary Figure 1 compares the nuclei size and proliferation rate of HUVEC cultures at passage 3 and 10. Supplementary Figure 2 compares the phenotype and the behavior of human fibroblasts at early and late passages. Supplementary Figure 3 describes the gene expression analysis (for the P16 and P21 mRNAs) performed in human fibroblasts at early and late passages. Supplementary Figure 4 shows the proliferation analysis using Ki67 as a marker for proliferation. Supplementary Figure 5 shows the effect of a chronic vitamin E supplementation in human fibroblasts during the progression of cellular senescence. [file 563247.f1.pdf]
